# Cooked Meats

**Published:** 1876-09

**Authors:** A. M. Ferris

**Affiliations:** N. Y. Manager, Wilson Packing Company; 137 Eighth Street, New York


In the August issue of the JOURNAL we published an article giving the results of an examination of the subject of the cooked meats, which are packed and sent abroad for use as food. Immediately on its appearance, orders came in for a much larger number of copies of the JOURNAL than we had on hand after supplying our subscribers and the various news agents. We have accordingly decided to re-publish the article in the present number. We do this the more willingly because the subject is one of very great importance, and because it affords us an opportunity to publish a note which we have received from the manager of the New York house. On the fifth day of August, several days after the publication of our August JOURNAL, the eminent chemist, Prof. R. Ogden Doremus, addressed a note to the Wilson Packing Company, giving the results of very careful examination of the canned meats of this Company, in which he says: "From general microscopical and chemical observation, I am of the opinion that the samples of corned beef are free from any organic, inorganic, or other impurity, and that they are palatable and healthful articles of diet." It thus appears that our conclusions are sustained by the highest chemical authority in the country. We subjoin the letter of Mr. Ferris:

NEW YORK, August 9, 1876.

TO THE EDITOR OF HALL'S JOURNAL OF HEALTH,

137 Eighth Street, New York:

DEAR SIR—We beg to thank you for the careful investigation which you have given to the subject of cooked meats as evinced by the editorial which appears in your August issue. We recognize the value of your judgment in all matters pertaining to health, and have no doubt that the public at large will be fully persuaded through your testimony that our meats are pure and wholesome and free from all taint or impurity. It shall be our duty to see that the high character which you assign to our products shall be fully maintained. With our large business—amounting to more than two and a half millions of dollars annually, and rapidly increasing—it would manifestly be a very short-sighted policy in us to pack poor meats or to use imperfect processes. On the contrary, should science develop any method by which better results could unquestionably be secured, we should hasten to adopt them.

We herewith inclose you our advertisement, which please insert in your September issue, to occupy one page, and send bill to us, charging your usual rates.

WILSON PACKING COMPANY,

A. M. FERRIS, N. Y. MANAGER.

